# Supplementary material for: MetaRibo-Seq measures translation in microbiomes
Source: Nat Commun. 2020 Jun 29;11:3268. doi: 10.1038/s41467-020-17081-z (PMC7324362; doi:10.1038/s41467-020-17081-z)
Supplement: Supplementary file 10 — Supplementary Data 7 [file 41467_2020_17081_MOESM10_ESM.zip › File2/Confidence_High_Taxonomy/11941_out.krona.html]

Javascript must be enabled to view this page.

members
magnitude
magnitudeUnassigned
count
unassigned
taxon
rank

11941\_out

5

5
2
superkingdom

5
1239
phylum

186801
5
class

order
5
186802

family
1
31979

1
1485
genus


SRS023715\_contig\_number\_2945
1
1262824
species

family
3
4

SRS043768\_contig\_number\_30330SRS045244\_contig\_number\_contig-100\_3400.3401SRS077730\_contig\_number\_11407
186803

841
1
genus

1

SRS149879\_contig\_number\_12979
1262941
species
